# A Comparative Study of Temperature Variations in Incisor Root Surfaces During Root Canal Preparation Using Various Rotary Systems and Irrigation Protocols

**DOI:** 10.3390/jcm13237484

**Published:** 2024-12-09

**Authors:** Mihai Paven, Adrian-George Marinescu, Osama Abuabboud, Laura-Elena Cirligeriu, Luminita Maria Nica, Vlad Tiberiu Alexa, Ruxandra Sava Rosianu, Atena Galuscan, Roxana Oancea

**Affiliations:** 1 Department of Restorative Dentistry and Endodontics, Research Center TADERP, Faculty of Dentistry, “Victor Babeș” University of Medicine and Pharmacy Timișoara, 300041 Timișoara, Romania; mihai.paven@umft.ro (M.P.); marinescu.adrian@umft.ro (A.-G.M.); osama.abuabboud@umft.ro (O.A.); cirligeriu.laura@umft.ro (L.-E.C.); 2 Translational and Experimental Clinical Research Centre in Oral Health, Department of Preventive, Community Dentistry and Oral Health, Faculty of Dentistry “Victor Babeș” University of Medicine and Pharmacy Timișoara, 300041 Timișoara, Romania; vlad.alexa@umft.ro (V.T.A.); sava-rosianu.ruxandra@umft.ro (R.S.R.); galuscan.atena@umft.ro (A.G.); roancea@umft.ro (R.O.)

**Keywords:** dentistry, endodontics, root canal therapy, thermography, tooth, nonvital

## Abstract

**Background/Objectives:** This study investigates the temperature changes on the external root surface during root canal preparation using different rotary systems and assesses the impact of irrigation temperatures. **Methods:** Sixty extracted human maxillary incisors were divided into four groups based on the rotary system used: ProTaper Next (Dentsply Sirona, Ballaigues, Switzerland), Reciproc Blue (VDW GmbH, Munich, Germany), WaveOne Gold (Dentsply Sirona, Ballaigues, Switzerland), and TruNatomy (Dentsply Sirona, Ballaigues, Switzerland). These systems differ in cutting efficiency and design. Temperature measurements during instrumentation and irrigation were recorded using a FLIR E60bx thermal camera (Teledyne FLIR LLC, Hudson, NH, USA). Irrigations were conducted with sodium hypochlorite at room temperature and heated to 60 °C. **Results:** The ProTaper Next group exhibited the highest average temperature increase during instrumentation (5.2 °C), followed by WaveOne Gold (4.3 °C), Reciproc Blue (3.7 °C), and TruNatomy (2.8 °C). During irrigation with heated sodium hypochlorite, temperature rises recorded were 6.8 °C for ProTaper Next, 5.9 °C for WaveOne Gold, 5.2 °C for Reciproc Blue, and 4.1 °C for TruNatomy. Statistical analysis revealed a significant negative correlation between dentin thickness and temperature rise, with r-values ranging from −0.62 to −0.87 across the groups. No significant correlation was found between canal diameter and temperature change, indicating that the canal’s size does not influence the thermal impact as much as the properties of the rotary system and irrigation temperature. **Conclusions:** Different rotary systems produce varying levels of temperature increases on the external root surface, influenced significantly by the thickness of the dentin and the temperature of the irrigation solution. These variations necessitate careful selection of instrumentation and irrigation protocols to minimize potential thermal damage to surrounding periodontal tissues.

## 1. Introduction

Root canal treatment is a fundamental procedure in endodontics aimed at eliminating infection and preserving natural teeth [1,2]. The success of endodontic therapy relies on thorough cleaning and shaping of the root canal system, which often involves the use of rotary instrumentation systems [3,4]. However, the mechanical action of these instruments can generate heat due to friction, potentially affecting the surrounding periodontal tissues [5,6].

Historically, the development of endodontic instruments has evolved significantly since the 17th century when Pierre Fauchard, known as the father of modern dentistry, laid the groundwork for dental practices [7]. The introduction of nickel-titanium (NiTi) rotary instruments revolutionized endodontic procedures by improving flexibility and efficiency compared to stainless steel instruments [8,9]. Various NiTi systems with different designs and motions have been developed to enhance cutting efficiency and reduce procedural errors [10,11].

Despite the advantages of rotary NiTi instruments, concerns have been raised regarding the heat generated during canal preparation [12,13]. Studies have indicated that excessive temperature increases can cause damage to periodontal tissues, including bone resorption, necrosis, and ankylosis [14,15]. Eriksson and Albrektsson demonstrated that a temperature rise of 10 °C can lead to periodontal ligament damage and bone resorption [5].

In addition to the mechanical parameters of torque and speed, the introduction of lubricants in the use of rotary instruments warrants consideration [16]. While it is clear that the fluid itself does not prevent the tooth from overheating, lubricants play a crucial role in reducing friction at the interface between the instrument and the tooth structure. This reduction in friction not only minimizes the generation of heat during instrument rotation but also potentially enhances the lifespan of the instruments by decreasing wear and tear [17]. Consequently, effective lubrication could significantly mitigate the risk of thermal damage to periodontal tissues, an important consideration given the concerns surrounding the heat generated during root canal preparation.

Moreover, irrigation protocols, particularly the use of heated sodium hypochlorite solutions, have been employed to enhance disinfection efficacy [18,19]. However, the use of heated irrigants may further contribute to temperature increases on the external root surface, raising concerns about potential thermal injury to periradicular tissues [20,21].

The thickness of the root dentin plays a crucial role in insulating the external root surface from the heat generated within the canal [22,23]. Teeth with thinner dentin walls may be at greater risk of transmitting heat to the surrounding tissues [24]. Therefore, understanding the relationship between dentin thickness, instrumentation systems, and temperature changes is essential for safe endodontic practice.

The null hypothesis of the study is that there is no significant difference in the temperature changes on the external root surfaces during root canal preparation when different rotary systems are used and under varying irrigation temperatures. Therefore, this study aims to evaluate the temperature changes on the external root surface of extracted human teeth during root canal preparation using different rotary systems and during irrigation with sodium hypochlorite at different temperatures.

## 2. Materials and Methods

### 2.1. Tooth Selection and Preparation

The current study was developed at the Faculty of Dentistry of the Victor Babes University of Medicine and Pharmacy in Timisoara, Romania. Sixty extracted human maxillary incisors with fully developed apices were collected for this in vitro study. Teeth were selected based on the absence of root caries, cracks, resorptions, or previous endodontic treatment, verified by visual inspection and radiographic examination. The teeth were stored in distilled water until use to prevent dehydration.

Access cavities were prepared using high-speed diamond burs under water cooling. The working length was determined by inserting a size 10 K-file into the canal until it was visible at the apical foramen and then subtracting 1 mm. The coronal portion of each tooth was adjusted to standardize the working length at 17 mm. Teeth were embedded vertically in silicone impression material (Silaxil, Lascod, Italy) to simulate periodontal support and facilitate handling during instrumentation and imaging.

We specifically chose human maxillary incisors because of their relatively uniform size and shape, which minimizes the variability that could potentially confound the results. Additionally, incisors typically have a single canal, making them ideal for standardized treatment in studies focusing on endodontic procedures. This choice helps to maintain consistency in the handling and instrumentation across all samples, thus providing more reliable and generalizable data.

In this study, the teeth selected were indicated for extraction primarily due to periodontal disease. These teeth retained enough structural integrity for experimental use, allowing us to maintain a controlled study environment and ensuring the results accurately reflected the effects of the variables tested. This approach minimized confounding factors associated with decay or significant structural damage.

### 2.2. Instrumentation Protocols

For this study, we determined the sample size required to achieve sufficient statistical power by considering factors such as expected effect size, standard deviation from preliminary data, and an alpha level of 0.05. Based on these parameters, we concluded that a total of 60 samples would allow us to detect significant differences with an appropriate level of power. The samples were evenly distributed into four groups of 15 teeth each to ensure comparable and robust analyses across the different rotary systems being tested. The teeth were randomly assigned by computer-generated random numbers into four groups (n = 15) based on the rotary instrumentation system used.

Group 1 (ProTaper Next): Canals were prepared using the ProTaper Next system (Dentsply Sirona, Ballaigues, Switzerland), instruments X1 17/.04 and X2 25/.06. These instruments feature a variable taper design that enhances cutting efficiency and flexibility. A reproducible glide path was established using a ProGlider instrument.

Group 2 (Reciproc Blue): Canals were prepared with a single Reciproc Blue R25 instrument (25/.08) produced by VDW GmbH, Munich, Germany. This system utilizes a single-file reciprocating motion that simplifies the procedure by eliminating the need for multiple files and reducing the risk of file fatigue and breakage.

Group 3 (WaveOne Gold): Canals were prepared using the WaveOne Gold system (Dentsply Sirona, Ballaigues, Switzerland), instruments Small 20/.07 and Primary 25/.07. Similar to Reciproc Blue, these files operate in a reciprocating motion but are designed with a gold metallurgy that increases flexibility and resistance to cyclic fatigue.

Group 4 (TruNatomy): Preparation was completed using TruNatomy instruments (Glider 17/.02v, Small 20/.04v, and Prime 26/.04), designed by Dentsply Sirona, Ballaigues, Switzerland, for minimally invasive preparation to preserve more dentinal structure. The system emphasizes conservative dentin removal and efficient debris evacuation.

Each group’s instrumentation was carefully standardized and performed by a single operator using an endodontic motor set to the specifications recommended by each manufacturer. Irrigation between each instrumentation step involved 2 mL of 5.25% sodium hypochlorite, ensuring consistent cleaning and disinfection across all groups. This methodical approach provided a controlled environment to assess and compare the thermal effects of different rotary systems during root canal treatment.

### 2.3. Thermal Imaging and Temperature Measurement

Thermal imaging was used to monitor temperature variations, and the effect of dentin thickness on temperature transmission was assessed. Temperature changes on the external mesial root surface were recorded using a FLIR E60bx thermal imaging camera (Teledyne FLIR LLC, Wilstonville, OR, USA). The camera was positioned at a fixed distance to capture the entire root surface. Baseline temperature readings were taken before instrumentation.

During canal preparation, continuous thermal imaging was performed to record real-time temperature changes. The maximum temperature reached on the external root surface in the coronal, middle, and apical thirds was noted for each tooth.

**Figure 1:**
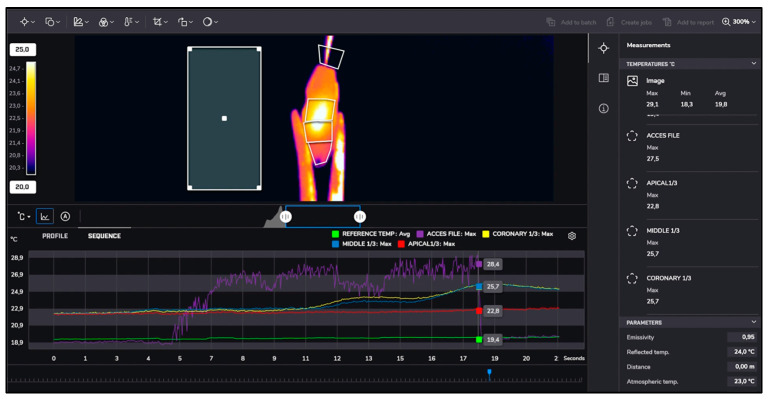
Reciproc Blue measurements.

**Figure 2:**
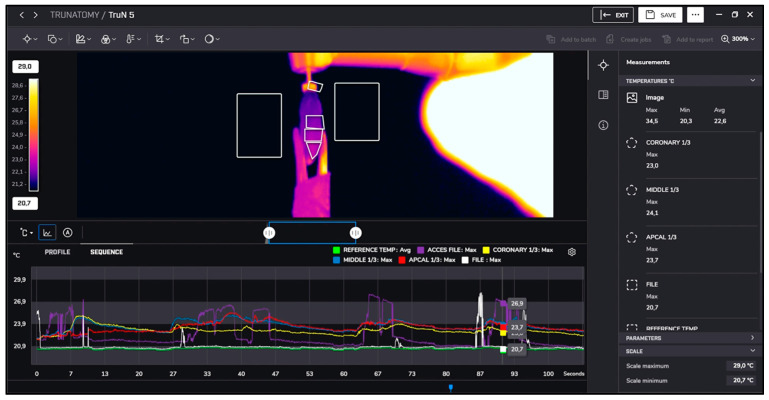
TruNatomy measurements.

**Figure 3:**
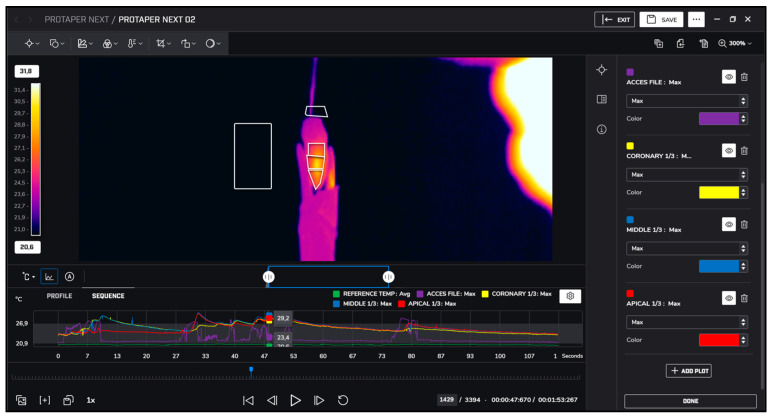
ProTaper Next measurements.

**Figure 4:**
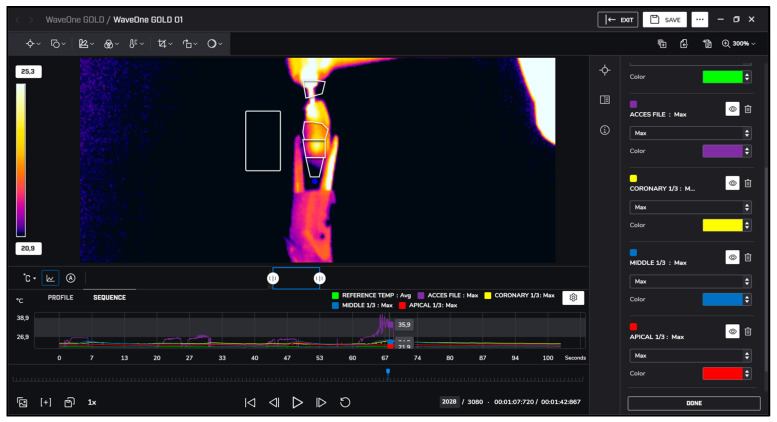
WaveOne Gold measurements.

After instrumentation, irrigation was performed with 5.25% sodium hypochlorite at room temperature (approximately 22 °C) and heated to 60 °C. Temperature changes during irrigation were recorded similarly using thermal imaging, as presented in Figure 1, Figure 2, Figure 3 and Figure 4.

### 2.4. Data Analysis

**Figure 5:**
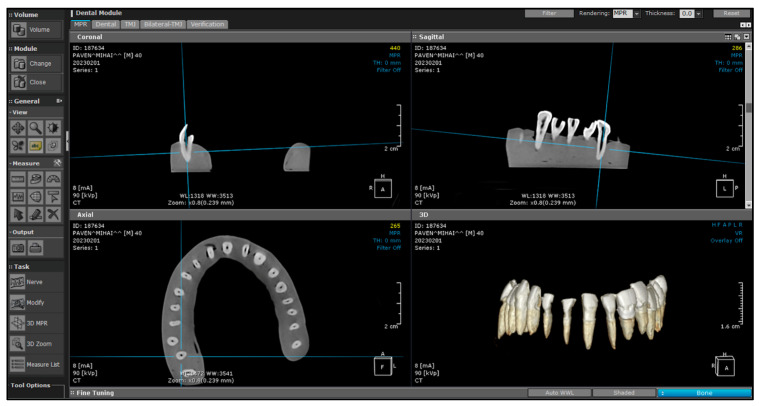
CBCT Scan.

Cone-beam computed tomography (CBCT) scans were taken before and after instrumentation to measure the thickness of the root dentin on the mesial surface and the diameter of the root canal in the coronal, middle, and apical thirds. The CBCT scans were performed using the Carestream CS 9600 system, manufactured by Carestream Dental LLC, Atlanta, GA, USA. These measurements were carried out using the CS Imaging software version 8, included with the Carestream CBCT unit, ensuring accurate and reproducible results. This system offers high-resolution imaging with a slice thickness of 90 μm, which is ideal for detailed analysis of dental structures (Figure 5).

### 2.5. Statistical Analysis

The statistical analysis was conducted using IBM SPSS Statistics software, version 26. Data were statistically analyzed using analysis of variance (ANOVA) to compare temperature changes among the groups. Post hoc Tukey tests were used for pairwise comparisons. Pearson correlation coefficients were calculated to assess the relationship between dentin thickness and temperature rise. A *p*-value of less than 0.05 was considered statistically significant.

## 3. Results

Statistical analysis using one-way ANOVA revealed significant differences among the groups in all regions (*p* < 0.001). The ProTaper Next group exhibited the highest mean dentin thickness across all root thirds, with values of 1.61 mm (coronal), 1.34 mm (middle), and 1.02 mm (apical). The Reciproc Blue and WaveOne Gold groups displayed intermediate thickness values, while the TruNatomy group consistently showed the lowest mean dentin thickness, measuring 1.10 mm (coronal), 0.95 mm (middle), and 0.75 mm (apical).

Standard deviations indicated the variability within each group. The ProTaper Next group had a higher SD in the coronal third (0.42 mm), suggesting more variation in dentin thickness among samples in that group. In contrast, the TruNatomy group displayed lower SD values across all thirds, indicating more uniform dentin thickness among its samples. These differences in dentin thickness are critical because dentin acts as an insulating barrier against heat transmission. Thinner dentin walls, as seen in the TruNatomy group, may allow more heat to reach the external root surface during instrumentation and irrigation, potentially increasing the risk of thermal damage to periodontal tissues. The significant *p*-values from ANOVA (<0.001) confirmed that the variations in dentin thickness among the groups were statistically significant. Post hoc Tukey tests further indicated that the TruNatomy group differed significantly from the other groups (*p* < 0.001), emphasizing its minimally invasive design that preserves more tooth structure internally while resulting in thinner external walls (Table 1).

The temperature increases were measured in the coronal, middle, and apical thirds of the roots. Statistical analysis showed significant differences among the groups in all regions (*p* < 0.001). The ProTaper Next group demonstrated the highest mean temperature increases, with 5.0 °C in the coronal third, decreasing to 3.0 °C in the apical third. This trend reflects the greater friction and heat generation in the coronal portion due to larger instrument diameters and more extensive dentin contact. The Reciproc Blue and WaveOne Gold groups showed moderate temperature increases, while the TruNatomy group exhibited the lowest increases, with mean values of 2.5 °C (coronal), 1.8 °C (middle), and 1.2 °C (apical).

Standard deviations were relatively low across all groups, indicating consistent results within each group. The decreasing temperature trend from coronal to apical thirds across all groups may be attributed to the natural tapering of the root canal, leading to reduced instrument contact and friction in the apical region. Post hoc Tukey tests confirmed significant differences between the ProTaper Next group and the TruNatomy group (*p* < 0.001) in all root thirds. Additionally, significant differences were found between the ProTaper Next group and the other groups (Reciproc Blue and WaveOne Gold), though these differences were less pronounced (*p* < 0.05), as presented in Table 2.

Significant differences were found between the ProTaper Next and TruNatomy groups across all root thirds (*p* < 0.001), with mean temperature differences ranging from 1.8 °C (apical) to 2.5 °C (coronal). This indicates that the ProTaper Next system generates significantly more heat compared to the TruNatomy system, likely due to its design and continuous rotation motion. Comparisons between ProTaper Next and Reciproc Blue showed smaller mean differences (0.5 °C to 0.8 °C) but were still statistically significant (*p* < 0.05), suggesting that ProTaper Next generates more heat than Reciproc Blue. The differences between ProTaper Next and WaveOne Gold were slightly larger (0.8 °C to 1.2 °C) and also significant (*p* < 0.01).

Significant differences were also observed between Reciproc Blue and TruNatomy and between WaveOne Gold and TruNatomy (*p* < 0.001), indicating that the TruNatomy system consistently produced lower temperature increases compared to the other systems. No significant differences were found between Reciproc Blue and WaveOne Gold (*p* > 0.05), implying that these two reciprocating systems have similar thermal profiles during instrumentation (Table 3).

Table 4 summarizes the mean temperature increases on the external root surface during irrigation with sodium hypochlorite at room temperature and heated to 60 °C for each group. All groups exhibited significant increases in external root surface temperature when irrigated with heated sodium hypochlorite compared to room temperature irrigation (*p* < 0.001). The ProTaper Next group showed the highest mean temperature increase during heated irrigation (6.0 °C), resulting in a mean difference of 4.5 °C compared to room temperature irrigation. The TruNatomy group had the lowest temperature increases during both room temperature (0.8 °C) and heated irrigation (3.0 °C).

The substantial temperature increases during heated irrigation raise concerns about potential thermal damage to periodontal tissues. The ProTaper Next group’s temperature rises during heated irrigation approached levels that, when combined with the heat generated during instrumentation, could exceed thresholds associated with tissue injury. Clinically, while heated sodium hypochlorite can enhance disinfection efficacy, these findings suggest caution is warranted. The combination of instrument-generated heat and heated irrigants may cumulatively elevate temperatures to harmful levels, particularly in teeth with thinner dentin walls or compromised periodontal support (Table 4).

Table 5 presents the Pearson correlation coefficients between dentin thickness and maximum temperature increase during instrumentation for the coronal, middle, and apical thirds. The negative correlation coefficients indicate an inverse relationship, where thinner dentin walls are associated with higher temperature increases on the external root surface.

In the apical third, the strongest negative correlation was observed (r = −0.85, *p* < 0.001), suggesting that dentin thickness plays a significant role in insulating against heat transmission in this region. The middle (r = −0.82) and coronal (r = −0.78) thirds also showed strong negative correlations, all statistically significant (*p* < 0.001).

These results emphasize the importance of considering dentin thickness when assessing the risk of thermal injury during root canal preparation. Teeth with thinner dentin walls are more susceptible to higher external temperature increases due to reduced insulation, highlighting the need for careful instrument selection and thermal management in such cases (Table 5).

Table 6 compiles the maximum temperature increases observed during instrumentation and heated irrigation for each group, along with the combined maximum temperature increase. It also indicates whether the combined temperature increase exceeded the clinical threshold of 10 °C, above which thermal injury to periodontal tissues may occur.

The ProTaper Next group had the highest combined maximum temperature increase (12.6 °C), exceeding the 10 °C threshold. The Reciproc Blue group also exceeded the threshold with a combined increase of 10.5 °C. The WaveOne Gold group’s combined temperature increase was 9.5 °C, approaching the threshold. The TruNatomy group remained well below the threshold with a combined increase of 6.5 °C. These findings have significant clinical implications. Exceeding the 10 °C threshold raises concerns about potential damage to the periodontal ligament and surrounding bone. The results suggest that the use of the ProTaper Next system, especially in conjunction with heated irrigation, may pose a higher risk of thermal injury (Table 6).

## 4. Discussion

### 4.1. Interpretation of Findings

The present study evaluated temperature changes on the external root surface during root canal preparation using four different rotary instrumentation systems and during irrigation with sodium hypochlorite at different temperatures. The findings demonstrated that both the choice of instrumentation system and the temperature of the irrigant significantly influence external root surface temperatures, with potential implications for periodontal health. The ProTaper Next system generated the highest temperature increases during instrumentation, likely due to its continuous rotation motion and larger tapers, leading to increased frictional heat. In contrast, the TruNatomy system produced the lowest temperature increases, consistent with its minimally invasive design and smaller tapers.

Irrigation with heated sodium hypochlorite significantly increased external root surface temperatures across all groups. When combined with the heat generated during instrumentation, the total temperature increases in the ProTaper Next and Reciproc Blue groups exceeded the critical 10 °C threshold associated with thermal injury to periodontal tissues. The strong negative correlations between dentin thickness and temperature increases highlighted the role of dentin as an insulating barrier. Thinner dentin walls, particularly in the apical third, allow more heat to reach the external root surface, increasing the risk of thermal damage.

In a comparative analysis, the studies by Caiado et al. [25] and Kasuga et al. [13] explored temperature dynamics and mechanical responses in dental procedures under varying conditions, yielding insightful results for clinical practices. Caiado et al. [25] focused on the in vivo temperature fluctuations on root canal walls during the preparatory stages of endodontic post cementation. Their findings indicated significant temperature variations, with the highest temperature recorded at 35.5 ± 0.8 °C during canal space drilling and the lowest at 34.0 ± 0.9 °C following saline rinsing, highlighting the thermal impact of different procedural stages. Similarly, Kasuga et al. [13] examined the phase transformation and mechanical properties of heat-treated nickel-titanium rotary endodontic instruments at room (25 ± 1 °C) and body (37 ± 1 °C) temperatures. They found that instruments like EDM exhibited lower bending loads and higher cyclic fatigue resistance (NCF) than non-heat-treated instruments, such as Mtwo, with significant variations between the temperatures. Interestingly, the bending loads of heat-treated instruments were notably lower, and NCF was higher at both temperatures, but the difference was less pronounced at body temperature, suggesting that the thermal properties and mechanical behavior of endodontic materials can substantially influence clinical outcomes.

Similarly, Jamleh et al. [26] assessed the shaping ability of thermally treated files in simulated canals with double curvature, where they found that ZenFlex (ZF) groups shaped the canals faster than ProTaper Next (PTN) groups, with cooling seemingly reducing canal deviations, although not significantly. The maximum shaping torques were comparable among all groups, indicating that cooling did not affect the torque required. Similarly, Rajnics et al. [27] investigated temperature changes during navigated endodontic root canal preparation, demonstrating that access cavity preparation and the use of cool fluids significantly reduced temperature elevations. Their results showed the highest temperature increases occurred at higher drill speeds without prior access cavity preparation, with cooling markedly reducing these elevations. Notably, the coolest fluid temperatures (4–6 °C) resulted in the lowest temperature elevations, highlighting the critical role of cooling in preventing thermal damage.

Moreover, Yiğit et al. [28] investigated the impact of temperature on the cyclic fatigue resistance of NiTi endodontic files, finding that as the temperature increased, the number of cycles to fracture (NCF) significantly decreased for all files tested. Notably, the ROTATE files performed best at the extremes of the temperature spectrum (4 °C and 60 °C), while the HyFlex EDM showed the highest NCF at a moderate temperature of 35 °C. Similarly, Reynette et al. [29] explored how different endodontic motors affect the mechanical behavior of root canal shaping instruments, particularly under different motion settings. Their study revealed that the type of motor and its operational mode (continuous rotation vs. reciprocating motion) significantly influenced the instruments’ performance, with specific motors like the Jeni Motor enhancing the mechanical behavior of the instruments during reciprocating motion.

The study by Özkoçak et al. [30] examined the temperature increases on the external root surface during endodontic treatments using different single-file systems. Their results indicated that the OneShape file system produced the lowest temperature increases, while the WaveOne file exhibited the highest, though the differences between Reciproc and WaveOne were not statistically significant. In a similar manner, Savitha et al. [31] conducted a systematic review and meta-analysis to evaluate the effect of body temperature on the cyclic fatigue resistance of nickel-titanium (NiTi) endodontic instruments, finding a significant reduction in cyclic fatigue resistance at body temperature compared to room temperature. This suggests that endodontic instruments perform differently under varying thermal conditions, which can directly impact their durability and effectiveness during clinical use.

In a similar manner, the study by Dosanjh et al. [32] explored the impact of temperature on the cyclic fatigue of nickel-titanium rotary files, revealing distinct variations in the number of cycles to fracture (NCF) across different temperatures and file types. The Vortex Blue group exhibited a consistent decline in NCF when temperatures increased from 3 °C to 60 °C. Meanwhile, the ESX group’s NCF significantly dropped as the temperature rose to 37 °C, and the EdgeFile group saw an increase in NCF of up to 22 °C before experiencing a decrease as the temperature was further raised to 37 °C. In comparison, Madarati and Watts [33] investigated temperature rise (TR) on the external root surface during the removal of endodontic fractured instruments using Gates Glidden drills. Their findings indicated an increase in TR with both the size and speed of the drills, with the highest recorded TR being 10.85 °C at 8000 rpm with GG5 drills, which still remained below the threshold considered harmful for tissue damage.

Clinically, these findings underscore the importance of careful instrument selection and thermal management during endodontic procedures. Using systems that generate less heat, such as TruNatomy, and being cautious with heated irrigants can help minimize thermal risks. Preoperative assessment of dentin thickness and individualized treatment planning are essential for optimizing patient outcomes.

### 4.2. Study Limitations

The study presents several limitations that should be considered when interpreting the findings. First, it was conducted in vitro using extracted teeth, which may not replicate the dynamic biological and thermal conditions of in vivo settings where blood flow and tissue perfusion play a critical role in temperature modulation. Additionally, the sample size, although statistically sufficient, was limited to only 60 maxillary incisors, which might not reflect variations found in other types of teeth with different anatomical structures. Moreover, the focus on maxillary incisors alone limits the generalizability of the results to other tooth groups that might react differently under similar treatment conditions. Furthermore, the absence of a longitudinal component in the study design precludes understanding the long-term effects of the observed temperature increases, which is crucial for evaluating the clinical relevance of thermal changes on periodontal health.

For future research, it is imperative to extend these findings through in vivo studies, which would allow for the evaluation of thermal effects in a living system where biological factors can be accounted for. Expanding the types of teeth examined could also provide a broader understanding of how different anatomical features influence thermal response during endodontic procedures. Additionally, exploring the efficacy of various cooling methods or alternative irrigation solutions could offer insights into more effective strategies for controlling temperature elevations during root canal treatments. Long-term studies are needed to assess the clinical outcomes of these thermal changes, specifically looking at the potential for thermal damage to periodontal tissues over time and the effectiveness of different preventive measures. These studies could provide essential data to develop guidelines that optimize patient safety and treatment outcomes in endodontics.

## 5. Conclusions

The study conclusively found that different rotary instrumentation systems produce varying degrees of temperature increase on the external root surface during root canal preparation of human maxillary incisors. The ProTaper Next system consistently generated the highest temperature increases, while the TruNatomy system produced the lowest, illustrating significant differences in thermal impact among the systems tested. Additionally, irrigation with heated sodium hypochlorite significantly raised external root surface temperatures compared to room-temperature irrigation. In some instances, the cumulative effect of both instrumentation and heated irrigation exceeded the critical threshold of 10 °C, posing a potential risk of thermal injury to the surrounding periodontal tissues. An inverse relationship was also noted between dentin thickness and temperature increase, indicating that thinner dentin walls are more susceptible to higher external temperatures. These findings emphasize the need for dental clinicians to judiciously select their instrumentation and irrigation protocols, particularly when treating incisors, to minimize thermal risks. The use of minimally invasive systems and careful management of irrigation temperatures are recommended to maintain periodontal health during endodontic treatments.

## Figures and Tables

**Table 1:** Dentin thickness measurements in coronal, middle, and apical thirds.

Group (Min-Max)	Mean Coronal Dentin Thickness (mm) ± SD	Mean Middle Dentin Thickness (mm) ± SD	Mean Apical Dentin Thickness (mm) ± SD	*p*-Value *
ProTaper Next	1.61 ± 0.42 (1.19–2.03)	1.34 ± 0.35 (0.99–1.69)	1.02 ± 0.26 (0.76–1.28)	<0.001
Reciproc Blue	1.45 ± 0.38 (1.07–1.83)	1.28 ± 0.30 (0.98–1.58)	0.95 ± 0.24 (0.71–1.19)	
WaveOne Gold	1.35 ± 0.36 (0.99–1.71)	1.20 ± 0.28 (0.92–1.48)	0.88 ± 0.22 (0.66–1.10)	
TruNatomy	1.10 ± 0.18 (0.92–1.28)	0.95 ± 0.15 (0.80–1.10)	0.75 ± 0.12 (0.63–0.87)	

SD—Standard Deviation; *—Two-way ANOVA.

**Table 2:** Maximum temperature increases during instrumentation.

Group (Min-Max)	Mean Coronal ΔT (°C) ± SD	Mean Middle ΔT (°C) ± SD	Mean Apical ΔT (°C) ± SD	*p*-Value *
ProTaper Next	5.0 ± 0.8 (4.2–5.8)	4.0 ± 0.7 (3.3–4.7)	3.0 ± 0.6 (2.4–3.6)	<0.001
Reciproc Blue	4.2 ± 0.6 (3.6–4.8)	3.5 ± 0.5 (3.0–4.0)	2.5 ± 0.5 (2.0–3.0)	
WaveOne Gold	3.8 ± 0.5 (3.3–4.3)	3.1 ± 0.4 (2.7–3.5)	2.2 ± 0.3 (1.9–2.5)	
TruNatomy	2.5 ± 0.4 (2.1–2.9)	1.8 ± 0.3 (1.5–2.1)	1.2 ± 0.2 (1.0–1.4)	

SD—Standard Deviation; *—Two-way ANOVA.

**Table 3:** Pairwise comparisons of temperature increases between groups (Post Hoc Tukey Tests).

Comparison	Mean Difference Coronal ΔT (°C)	*p*-Value	Mean Difference Middle ΔT (°C)	*p*-Value	Mean Difference Apical ΔT (°C)	*p*-Value
ProTaper Next vs. TruNatomy	2.5	<0.001	2.2	<0.001	1.8	<0.001
ProTaper Next vs. Reciproc Blue	0.8	0.012	0.5	0.018	0.5	0.015
ProTaper Next vs. WaveOne Gold	1.2	0.005	0.9	0.007	0.8	0.006
Reciproc Blue vs. TruNatomy	1.7	<0.001	1.7	<0.001	1.3	<0.001
WaveOne Gold vs. TruNatomy	1.3	<0.001	1.3	<0.001	1	<0.001
Reciproc Blue vs. WaveOne Gold	0.4	0.075	0.4	0.082	0.3	0.09

**Table 4:** Temperature increases during irrigation with sodium hypochlorite.

Group	Mean ΔT Room Temp Irrigation (°C) ± SD	Mean ΔT Heated Irrigation (°C) ± SD	Mean Difference (°C)	Paired *t*-Test *p*-Value
ProTaper Next	1.5 ± 0.3	6.0 ± 0.7	4.5	<0.001
Reciproc Blue	1.2 ± 0.2	5.0 ± 0.6	3.8	<0.001
WaveOne Gold	1.0 ± 0.2	4.5 ± 0.5	3.5	<0.001
TruNatomy	0.8 ± 0.1	3.0 ± 0.4	2.2	<0.001

**Table 5:** Correlation between dentin thickness, canal diameter, and temperature increase during instrumentation.

Region	Pearson Correlation Coefficient for Dentin Thickness (r)	*p*-Value	Pearson Correlation Coefficient for Canal Diameter (r)	*p*-Value
Coronal	−0.78	<0.001	−0.05	0.626
Middle	−0.82	<0.001	−0.02	0.844
Apical	−0.85	<0.001	0.01	0.910

**Table 6:** Combined maximum temperature increases and clinical thresholds.

Group	Max ΔT Instrumentation (°C)	Max ΔT Heated Irrigation (°C)	Combined Max ΔT (°C)	Exceeds 10 °C Threshold?
ProTaper Next	5.8	6.8	12.6	Yes
Reciproc Blue	4.8	5.7	10.5	Yes
WaveOne Gold	4.3	5.2	9.5	Approaching
TruNatomy	3	3.5	6.5	No

## Data Availability

Data availability is subject to hospital approval.
